# Bacteriocin-encoding genes and ExPEC virulence determinants are associated in human fecal *Escherichia coli* strains

**DOI:** 10.1186/1471-2180-14-109

**Published:** 2014-04-28

**Authors:** Lenka Micenková, Barbora Štaudová, Juraj Bosák, Lenka Mikalová, Simona Littnerová, Martin Vrba, Alena Ševčíková, Vladana Woznicová, David Šmajs

**Affiliations:** 1 Department of Biology, Faculty of Medicine, Masaryk University, Kamenice 5, Building A6, Brno 625 00, Czech Republic; 2 Institute of Biostatistics and Analyses, Masaryk University, Kamenice 3, Building A1, Brno 625 00, Czech Republic; 3 Department of Clinical Microbiology, University Hospital Brno, Jihlavská 20, Brno 625 00, Czech Republic; 4 Department of Microbiology, Faculty of Medicine, Masaryk University and St. Anne's University Hospital, Pekařská 53, Brno 656 91, Czech Republic

**Keywords:** *Escherichia coli*, Colicin, Microcin, Bacteriocin, Virulence factor

## Abstract

**Background:**

A set of 1181 *E. coli* strains of human fecal origin isolated in the South Moravia region of the Czech Republic was collected during the years 2007–2010. Altogether, 17 virulence determinants and 31 bacteriocin-encoding genes were tested in each of them.

**Results:**

The occurrence of bacteriocin-encoding genes was found to be positively correlated with the occurrence of *E. coli* virulence factors. Based on the presence of virulence factors and their combinations, *E. coli* strains were classified as non-pathogenic *E. coli* (n = 399), diarrhea-associated *E. coli* (n = 179) and ExPEC strains (n = 603). Non-pathogenic and diarrhea-associated *E. coli* strains had a low frequency of bacteriocinogeny (32.6% and 36.9%, respectively). ExPEC strains encoding S-fimbriae (*sfa*), P-fimbriae (*pap*) and having genes for aerobactin biosynthesis (*aer*, *iucC*), α-hemolysis (*α-hly*) and cytotoxic necrosis factor (*cnf1*) were often bacteriocinogenic (73.8%), had a high prevalence of bacteriocin multi-producers and showed a higher frequency of genes encoding microcins H47, M, V, B17 and colicins E1, Ia and S4.

**Conclusions:**

The occurrence of bacteriocin-encoding genes and ExPEC virulence determinants correlate positively in *E. coli* strains of human fecal origin. Bacteriocin synthesis appears to modulate the ability of *E. coli* strains to reside in the human intestine and/or the virulence of the corresponding strains.

## Background

The endogenous human gut microbiome has several important functions including nourishment, the training of innate immunity and the regulation of epithelial development [1]. Although the *Escherichia coli* population represents a rather small portion of the intestinal bacterial microflora, *E. coli* nonetheless occupy an important niche with regard to their close proximity to intestinal epithelium, wherein they utilize available oxygen and facilitate anaerobic growth [2]. Intestinal microflora also prevent the growth of pathogenic bacteria, either by competing for nutrient sources, or through direct bacterial antagonism mediated by bacteriocins and bacteriophages [3].

*E. coli* is a highly diverse species with respect to its gene content, phenotype and virulence [4]. Based on different virulence factors, *E. coli* strains can be classified into three main groups: commensal, intestinal pathogenic and extraintestinal pathogenic *E. coli* (ExPEC) [5]. Commensal strains are commonly considered to be non-pathogenic. It has been shown that intestinal and extraintestinal pathogenic *E. coli* strains can develop from commensal strains by acquisition of virulence factors [6,7].

Intestinal pathogenic (diarrhea-associated) *E. coli* is a typical mucosal pathogen which uses different pathogenic strategies including invasion of host cells (enteroinvasive *E. coli*, EIEC), production of enterotoxins (enterotoxigenic *E. coli,* ETEC) and production of Shiga-like toxins (enterohemorrhagic *E. coli*, EHEC) [8]. Enteropathogenic *E. coli* (EPEC) strains cause attaching-and-effacing (A/E) lesions and harbor the EAF plasmid [8]. Diffuse-adherent strains of *E. coli* (DAEC) are characterized by continuous adherence to eukaryotic cells mediated by afimbrial adhesins [9], while entero-aggregative (EAggEC) strains produce an aggregative adherence (AA) pattern [10] when adhering to HEp-2 cells.

ExPEC strains carry different combinations of virulence factors. Johnson *et al.* (2003) defined ExPEC strains as those possessing 2 or more of the following virulence factors: P fimbriae, S/F1C fimbriae subunits, Dr-antigen binding adhesins, aerobactin receptor and group 2 capsule synthesis [11].

Another important characteristic of human *E. coli* strains is production of bacteriocins. Colicins and microcins are antimicrobial agents with a relatively narrow spectrum of activity [12-14]. In general, microcins are known to have a wider spectra of antibacterial activity compared to colicins [14,15]. Colicin Js [16,17] is unique in that it shares features of both colicins and microcins. The ecological role and molecular evolution of bacteriocinogeny are less clear but synthesis of bacteriocins may have both invasive and defensive functions in microbial communities [18]. Bacteriocin production is a common feature of many probiotic bacteria. For example, microcins H47 and M are active substances produced by the non-pathogenic, probiotic *E. coli* strain Nissle 1917 [19].

At the same time, several studies have shown an association between the production of some types of bacteriocins and pathogenic *E. coli* strains [20-23]. Previous studies have found that genes encoding colicin E1, as well as microcins H47, M, I47, E492 and V were associated with extraintestinal pathogenic *E. coli* strains [20-23]. Colicin E1 is also known to have toxic effects on eukaryotic cells and is considered to be a virulence factor in UPEC strains [21,24,25]. Microcins H47 and M are induced when iron is a limiting factor and are associated with competition for iron [22,26]. Synthesis of microcin H47 and M could therefore be advantageous in bacteremia of urinary tract origin [22,26].

Previously published studies have only provided partial insight into the association between bacteriocin production and bacterial virulence, as they were primarily focused upon UPEC strains and differed in the number of detected bacteriocin and virulence genes. Azpiroz *et al.* (2009) screened 5 microcin types in 125 UPEC strains and 9 virulence factors [20], while Budič *et al.* (2011) and Petkovšek *et al.* (2012) analyzed 14 virulence factors (primarily those associated with urinary tract infections) and 19 bacteriocin types in 105 UPEC strains [22,23]. Similarly, Abraham *et al.* (2012) analyzed 16 bacteriocin types and 18 virulence factors in a collection of 159 UPEC strains [27]. Together, these studies identified an association between microcins (M, H47, V, B17 and L) and several virulence genes [20,22,23,27]. Studies by Gordon *et al.* (2005) and Gordon and O’Brien (2006) focused on 266 fecal *E. coli* strains and identified an association between strains encoding at least one microcin type, and four genes involved in iron acquisition (from a total of 29 tested virulence determinants) [26,28].

The main aim of our study was to test the association between bacteriocin production and bacterial virulence within a large collection of *E. coli* strains (n = 1181) isolated from human gut microflora. In this study, new associations between bacteriocin-encoding genes and virulence determinants were found in human fecal *E. coli* strains.

## Results

### Detection of virulence determinants and bacteriocin-encoding genes

Altogether, 18 DNA determinants (pCVD432*, α-hly*, *afaI*, *aer*, *cnf1*, *sfa*, *pap*, *ial*, *lt*, *st*, *bfpA, eaeA, ipaH, iucC, fimA* and *stx1*, *stx2* and *ehly*) encoding 17 different virulence factors were tested in each of 1181 *E. coli* strains (Additional file 1: Table S1). The vast majority of strains (94.7%) possessed at least one virulence factor. The most common virulence determinant was the *fimA* gene (encoding fimbriae type I), which was detected in 87.9% of all strains.

Genes encoding 29 bacteriocin types including 24 colicin types (A, B, D, E1-9, Ia, Ib, Js, K, L, M, N, S4, U, Y, 5 and 10) and 5 microcin types (B17, C7, J25, L and V) were tested in each of 642 *E. coli* bacteriocin producer strains. Further, the prevalence of chloroform sensitive microcins H47 and M [19] was tested in each of the 1181 *E. coli* strains. The average prevalence of bacteriocinogeny in the set of 1181 *E. coli* strains was 54.4% (Additional file 1: Table S1). In contrast to other bacteriocin determinants, genes encoding colicins A, E4, E9 and L were not detected in any producer strain. Most of bacteriocin producers were strains producing two or more bacteriocin types (Additional file 1: Table S1).

### Association between bacteriocin and virulence determinants

**Figure 1:**
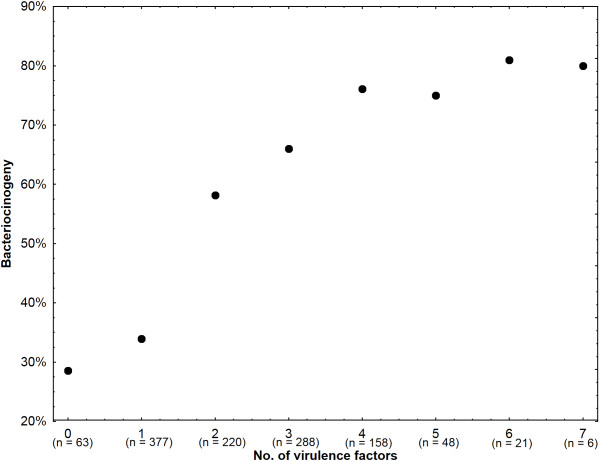
**Association between number of virulence factors encoded by *E. coli *strains and bacteriocin production.** Frequency of bacteriocinogeny in *E. coli* strains correlates with number of virulence factors coded by *E. coli.* The x axis represents the number of virulence factors coded by *E. coli* strains (n represents the number of strains encoding the appropriate number of virulence factors) and the y axis shows the frequency of bacteriocinogeny.

We found that 28.6% of *E. coli* strains possessing no virulence determinant (n = 63) produced bacteriocins and 34% of the strains harboring one virulence determinant (n = 377) produced bacteriocins. In addition, 58.2% of *E. coli* encoding two virulence determinants (n = 220) had bacteriocin genes and 70.6% of the strains with 3 to 7 virulence determinants (n = 521) were bacteriocinogenic (Figure 1).

**Figure 2:**
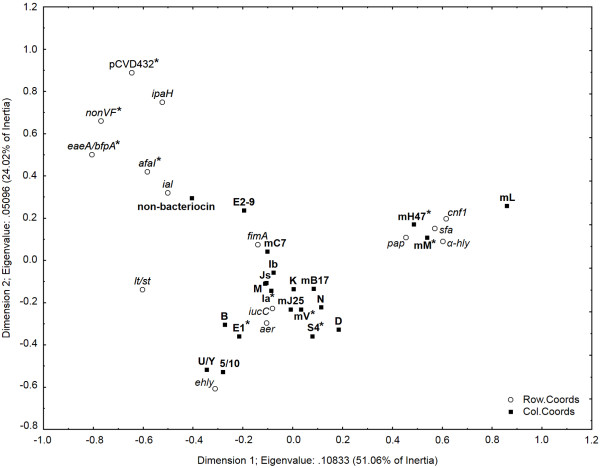
**Correspondence analysis for bacteriocin types and virulence factors.** Association between virulence factors (*α*-*hly*, *afaI*, *aer*, *cnf1*, *sfa*, *pap*, pCVD432, *ial*, *lt*, *st*, *bfpA*, *eaeA*, *ipaH*, *iucC*, *fimA*, *ehly*) and bacteriocin types (B, D, E1, E2-9, Ia, Ib, Js, K, M, N, S4, U/Y, 5/10, mB17, mC7, mH47, mJ25, mL, mM and mV) in 1181 *E. coli* strains. The x axis accounted for 51.06% of total inertia and the y axis for 24.02%. Please note the close association between virulence determinants *pap*, *sfa*, *cnf1* and *α-hly* and genes for microcins H47, M and L. Genes encoding aerobactin synthesis plotted close to bacteriocin genes encoding microcins J25, V and B17 and colicins B, E1, 5/10, Ia, Ib, Js, M, K, N, S4 and D. Fisher’s exact test was used to analyze the degree of association among bacteriocin types and virulence factors; statistically significant results for different virulence factors and bacteriocin types are indicated by asterisks (*α*-*hly*, *cnf1*, *sfa*, *pap –* mH47 and mM; *iucC*, *aer* – E1, Ia, S4 and mV; *afaI*, *eaeA/bfpA*, pCVD432, nonVF *–* bacteriocin non-producers).

A correspondence analysis (CA) was performed using individual virulence determinants and bacteriocin-encoding genes (Figure 2). In addition to this two-dimensional representation, Fisher’s exact test was used to analyze the association between bacteriocin types and virulence determinants. Genes encoding aerobactin synthesis were (*aer, iucC*) were significantly associated with genes for microcin V (p < 0.01) and with genes encoding colicins E1 (p < 0.01), Ia (p < 0.01) and S4 (p = 0.01). The *α-hly*, *cnf1*, *sfa* and *pap* virulence determinants were plotted together and were associated with genes for microcins H47 (p < 0.01) and M (p < 0.01). Bacteriocin non-producers were associated with *afaI* (p < 0.01), *eaeA/bfpA* (p < 0.01), pCVD432 (p = 0.03) and with strains in which virulence determinants were not detected (p < 0.01) (Figure 2).

### Association between bacteriocin-encoding genes and *E. coli* pathotypes

Based on the presence of virulence factors, *E. coli* strains were divided into three groups: (1) non-pathogenic (commensal, non enterovirulent, nonEVEC) *E. coli* (n = 399), (2) diarrhea-associated *E. coli* (EAggEC, ETEC, EIEC, EPEC and DAEC; n = 179) and (3) fecal *E. coli* with characteristics similar to ExPEC, denoted ExPEC in this study (n = 603) (Table 1). Non-pathogenic *E. coli* were defined as those with no detected genes for virulence factors or those that only had the gene for fimbriae type I (*fimA* gene). Diarrhea-associated *E. coli* strains encoded virulence factors typical for each of the diarrhea-associated pathotypes including EAggEC (pCVD432), ETEC (*lt*/*st*), EIEC (*ial*/*ipaH*), EPEC (*eaeA/bfpA*), EHEC (*stx1/stx2/ehly*) and DAEC (*afaI*) strains. All other strains containing genes for different virulence factors (e.g. α-hemolysin, P-fimbriae, S-fimbriae, cytotoxic necrosis factor, aerobactin synthesis) and combinations thereof were classified as ExPEC. The results of the correspondence analysis of individual virulence determinants and bacteriocin genes (Figure 2) showed that a majority of bacteriocin genes overlap with virulence determinants belonging to ExPEC strains.

**Table 1:** **Occurrence of virulence factors in *E. coli *** **pathotypes**

**Virulence factors**		**Pathotype**		
		Non-pathogenic E. coli*	Diarrhea-associated E. coli**	ExPEC***
		**n = 399 (%)**	**n = 179 (%)**	**n = 603 (%)**
Aggregative adherence plasmid	pCVD432	-	13 (7.3)	-
Invasive associated locus	*ial*	-	44 (24.6)	-
Heat-stable enterotoxin	*st*	-	8 (4.5)	-
Heat-labile enterotoxin	*lt*	-	7 (3.9)	-
Intimin	*eaeA*	-	26 (14.5)	-
Bundle-forming fimbriae	*bfpA*	-	1 (0.6)	-
Invasion plasmid H	*ipaH*	-	19 (10.6)	-
Aerobactin synthesis	*aer*	-	68 (38.0)	342 (56.7)
Fimbriae type 1	*fimA*	336 (84.2)	149 (83.2)	553 (91.7)
α-hemolysin	*α-hly*	-	3 (1.7)	88 (14.6)
Afimbrial adhesin	*afaI*	-	78 (43.6)	-
Aerobactin synthesis	*iucC*	-	80 (44.7)	396 (65.7)
Cytotoxic necrotizing factor	*cnf1*	-	1 (0.6)	43 (7.1)
S-fimbriae	*sfa*	-	6 (3.4)	227 (37.6)
P-fimbriae	*pap*	-	19 (10.6)	201 (33.3)
Shiga-toxin 1	*stx1*	-	-	-
Shiga-toxin 2	*stx2*	-	-	-
Enterohemolysin	*ehly*	-	9 (5.0)	-

*E. coli strains with no detected genes for virulence factors or those possessing only gene for fimbriae type I (*fimA* gene).

**EAggEC - pCVD432 (aggregative adherence plasmid); ETEC - *lt*/*st* (heat-labile and heat-stable enterotoxin); EIEC - *ial*/*ipaH* (invasion associated locus/invasion plasmid H); EPEC – *bfpA*/*eaeA* (bundle-forming fimbriae/intimin); EHEC (*stx1/stx2/ehly*); DAEC – *afaI* (afimbrial adhesin I).

***E. coli strains which contain different virulence factors (except those that are typical for previously described diarrhea-associated *E. coli*) typical for extraintestinal *E. coli* strains (α-hemolysin, P-fimbriae, S-fimbriae, cytotoxic necrosis factor, aerobactin synthesis).

The occurrence of bacteriocinogeny (i.e. occurrence of at least one bacteriocin-encoding gene) in nonEVEC strains (32.6%) and in diarrhea-associated *E. coli* strains (36.9%) was significantly lower than among ExPEC (73.8%; p < 0.01) (Table 2). In addition, a similar frequency of bacteriocin types was also found in both groups of nonEVEC and diarrhea-associated *E. coli*. Among nonEVEC strains, those with a single bacteriocin gene were most common, while ExPEC strains more often contained several bacteriocin genes in a single strain. Compared to nonEVEC and diarrhea-associated strains, ExPEC had higher frequencies of genes encoding microcins V, H47, M (p < 0.01 against both nonEVEC and diarrhea-associated strains) and gene encoding colicin E1 (p < 0.01 against nonEVEC, p = 0.04 against diarrhea-associated strains). In addition, compared to nonEVEC strains, ExPEC had higher frequencies of genes encoding microcin B17 (9.5%; p < 0.01) and colicins Ia (20.7%; p < 0.01), E1 (15.6%; p < 0.01) and S4 (1.8%; p = 0.01).

**Table 2:** **Occurrence of bacteriocinogeny and bacteriocin types among *E. coli *** **strains**

**Bacteriocinogeny**	**Pathotype**			Statistics*		
	**1. Non-pathogenic *E. coli***	**2. Diarrhea-associated *E. coli***	**3. ExPEC**	**1 x 2**	**1 x 3**	**2 x 3**
	**n = 399 (%)**	**n = 179 (%)**	**n = 603 (%)**	**p**	**p**	**p**
**Bacteriocinogenic strains**	130 (32.6)	66 (36.9)	445 (73.8)	-**	< 0.01	< 0.01
**Bacteriocin types**						
mV	18 (4.5)	18 (10.1)	152 (25.2)	0.04	< 0.01	< 0.01
mM	17 (4.3)	7 (3.9)	123 (20.4)	-	< 0.01	< 0.01
mH47	28 (7.0)	14 (7.8)	165 (27.4)	-	< 0.01	< 0.01
mB17	10 (2.5)	8 (4.5)	57 (9.5)	-	< 0.01	-
Ia	53 (13.3)	23 (12.8)	125 (20.7)	-	< 0.01	-
E1	19 (4.8)	15 (8.4)	94 (15.6)	-	< 0.01	0.04
S4	-	-	11 (1.8)	-	0.01	-
**Bacteriocin producer strains**						
Mono-producers***	63 (48.5)	23 (34.8)	141 (31.7)	-	< 0.01	-
Ia	23 (17.7)	3 (4.5)	18 (4.0)	0.04	< 0.01	-
Double-producers****	44 (33.8)	25 (37.9)	161 (36.2)	-	-	-
mH47, mM	5 (3.8)	4 (6.1)	50 (11.2)	-	0.03	-
Multi-producers*****	21 (16.2)	15 (22.7)	139 (31.2)	-	< 0.01	-

*Fisher’s exact test with Bonferroni correction.

**not statistically significant.

***producers of one bacteriocin type.

****producers of two bacteriocin types.

*****producers of three and more bacteriocin types.

## Discussion

In this study, the average prevalence of bacteriocinogenic *E. coli* strains isolated from fecal microflora was 54.4%. This value is close to the upper range limit seen in previous studies, where the prevalence of bacteriocinogenic *E. coli* strains varied from 25 to 55% [15,21,26,27,29-31]. However, these studies differed in a number of important ways including cultivation conditions and indicator bacteria used for detection of bacteriocin production and/or in the number of detected bacteriocin genes. Older studies on the prevalence of bacteriocinogeny in fecal *E. coli* strains only focused on the identification of colicin production [30,32]. While Šmarda and Obdržálek (2001) used five different indicator strains to detect colicin production in the fecal *E. coli* strain 1043 [32], Achtman *et al.* (1983) used 2 indicator strains for the detection of colicin producers in a sample of 234 fecal *E. coli* strains [30]. More recently, Gordon and O’Brien (2006) used PCR with 19 bacteriocin genes to screen 266 fecal *E. coli* strains (38% of which were bacteriocinogenic) [26], and Šmajs *et al.* (2010) detected 29 bacteriocin types in 411 fecal *E. coli* strains (55% of which were bacteriocin-encoding strains) [21].

Our results have revealed that the frequency of bacteriocinogeny in *E. coli* strains positively correlates with the detected number of virulence determinants. Bacteriocinogeny increased by as much as 75–80% depending on the number of encoded virulence factors. To our knowledge, this is the first time that the correlation between bacteriocinogeny frequency and the number of encoded virulence factors has been shown. This finding suggests that at least some bacteriocin-encoding genes should be considered as factors which increase the virulence of *E. coli* strains.

*E. coli* strains encoding only fimbriae type I did not show differences in the frequency of bacteriocinogeny compared to strains without genes for virulence factors. The role of fimbriae type I in development of human infections is not clear. Although deletion of the *fim* gene cluster from virulent *E. coli* strain O1:K1:H7 attenuated virulence in the urinary tract infection (UTI) model [33]; possession of fimbriae type 1 in *E. coli* strains from different sources was not found to correlate with the ability to cause UTIs [34-39]. Several virulence factors, typical for diarrhea-associated *E. coli* strains, including pCVD432 (EAggEC), *ial*/*ipaH* (EIEC), *eaeA/bfpA* (EPEC) and *afaI* (DAEC) were not found to be associated with bacteriocin genes. Bacteriocin producers therefore appear to be mainly associated with ExPEC virulence factors (*E. coli* strains containing combinations of *sfa*, *pap*, *aer*, *iucC*, *cnf1*, *α-hly* determinants). The occurrence of these virulence factors were associated with both chromosomally (microcins H47 and M) and plasmid encoded colicin (E1, Ia and S4) and microcin types (B17, V).

Presently, several bacteriocins including colicin E1, and microcins H47, I47, E492, M, and V are considered virulence factors in extraintestinal pathogenic *E. coli* strains [20-23]. Azpiroz *et al.*[20] and Budič *et al.*[22] found an association between production of microcins H47, I47, E492, M, and V and the distribution of virulence factors (i.e. *hlyA*, *cnf1*, *usp*, *iroN*, *iroCD*, *fyuA*, *papC*, *papG* and *tcpC*) in uropathogenic strains of *E. coli*; from these results they assumed that production of these bacteriocin types could contribute to development of bacteraemia. Although different sets of virulence determinants and bacteriocin genes were used in these studies, our findings match with these observations.

We also found these associations between bacteriocin production and ExPEC virulence determinants among human fecal *E. coli* isolates. Moreover, we have found new associations between 3 bacteriocin types (microcin B17, colicins Ia and S4) and the ExPEC virulence characteristics of human fecal *E. coli* strains. Given that colicin Ia and microcin B17 are known to be encoded on relatively large plasmids, it is possible that the association with more virulent strains is due to other genes being harbored on these plasmids, and not by colicin synthesis itself. However, colicin S4 was found to be encoded on a small plasmid (7.4 kb) [40] that was similar to the colicin E1-encoding plasmid (6 kb) [21]. Since these small plasmids do not encode virulence factors, colicin S4 appears to be a potentially important virulence factor and/or an important factor of resident *E. coli* strains.

The presence of virulence determinants (e.g. genes encoding P-fimbriae, siderophore aerobactin, hemolysin and expression of O antigens, which are typical for ExPEC strains; and capsular types K1 and K5) are associated with resident *E. coli* strains [41-44]. At the same time, ExPEC strains causing extraintestinal infections like urinary tract infections and sepsis/meningitidis are believed to originate from the gut microflora. Their virulence determinants including adhesins (P-fimbriae, S-fimbriae), toxins (e.g. hemolysin, cytotoxic necrotizing factor) and siderophores (e.g. aerobactin) appear to be important for *E. coli* strains to survive in the extraintestinal environment [45-47].

On the other hand, we found that diarrhea-associated strains from our set of 1181 fecal *E. coli* had a lower prevalence of bacteriocinogeny and a lower frequency of several bacteriocin producers. In addition, no specific bacteriocin types appear to be associated with virulence determinants that are typical for these strains. Unlike fecal strains which have the characteristics of ExPEC strains, diarrhea-associated strains are not considered to be resident human *E. coli* strains, which may explain the lower prevalence of bacteriocin genes.

In summary, bacteriocin synthesis is linked to strains with ExPEC characteristics and appears to increase the ability of *E. coli* to reside in the human gut. Moreover, at least several bacteriocin-encoding genes should be also considered as factors which increase the virulence of ExPEC strains.

## Conclusions

The frequency of bacteriocin-encoding genes was found to be positively correlated with the frequency of *E. coli* virulence determinants. *E. coli* with virulence characteristics of ExPEC strains, i.e. strains encoding virulence factors S-fimbriae (*sfa*), P-fimbriae (*pap*), cytotoxic necrosis factor (*cnf1*), α-hemolysin (*α-hly*) and aerobactin biosynthesis (*aer*, *iucC*) were more often found to harbor genes encoding synthesis of microcins (H47, M, V and B17) and colicins (E1, Ia and S4) than other tested *E. coli* strains.

## Methods

### Bacterial strains

*E. coli* strains were isolated from the intestinal microflora of 1181 patients living in South Moravia, Czech Republic. A set of 183 *E. coli* strains was isolated at St. Anne's University Hospital, Brno, CZ, and 998 *E. coli* strains at the University Hospital, Brno, CZ. *E. coli* strains were isolated between July 2007 and April 2010. 565 *E. coli* strains were isolated from female patients and 616 *E. coli* strains from males. All clinical samples were collected after patients gave informed consent. For children under the age of 18, consent was obtained from parents. The study was approved by the ethics committee of the Faculty of Medicine, Masaryk University, Brno, CZ. A single isolate of *E. coli* was collected from each patient. Testing with ENTEROtest16 (Erba Lachema, Czech Republic) was used for bacterial identification. Indicator strains used for screening of bacteriocin production and the control bacteriocin producers used for PCR detection of bacteriocin genes, were previously described in detail [21].

### Screening of bacteriocin production

Bacteriocin production was detected using the method described by Šmajs *et al.* (2010) [21]. Briefly, each of 1181 *E. coli* strains were simultaneously cultivated (37°C for 48 hours) in parallel on four different agar plates containing (i) TY (Trypton-yeast) agar (HiMedia, Mumbai, India) (1.5%, w/v, solid agar), (ii) Difco™ Nutrient broth (Difco Laboratories, Sparks, MD, USA), (iii) TY agar supplemented with mitomycin C, and (iv) TY agar supplemented with trypsin. Macrocolonies were then killed using chloroform vapors and overlaid with a top TY agar layer (0.7%, w/v, soft agar) containing 10^7^ cells from one of 6 indicator strains (*E. coli* K12-Row, *E. coli* C6 (φ), *E. coli* 5 K, *E. coli* P400, *E. coli* S40 and *Shigella sonnei* 17). The plates were subsequently incubated at 37°C for 24 hours and bacteriocin producers were identified.

### PCR detection of genes encoding bacteriocins

Detection of the 24 colicin and 7 microcin genes was carried out using the method described by Šmajs *et al.* (2010) [21]. Briefly, genomic DNA was isolated using DNAzol reagent (Invitrogen, Carlsbad, CA, USA) according to the manufacturer’s protocol. Template DNA was diluted 100-fold in sterile distilled water. All producer strains were tested, in parallel, using the colony PCR method (one bacterial colony from each strain was resuspended in 100 μl of sterile distilled water; then 1 μl of this suspension was added to the PCR mix). PCR reactions were performed using the primers described by Šmajs *et al.* (2010) [21]; for colicins E1, L and microcin M additional primer pairs were used (Additional file 2: Table S2). The following protocol was used for PCR amplification: 94°C (2 minutes); 94°C (30 seconds), 60°C (30 seconds), 72°C (1 minute), 30 cycles; 72°C (7 minutes). For colony PCR, the initial step was 5 minutes. Microcins H47 and M are sensitive to chloroform vapors [19], therefore all 539 bacteriocin-nonproducing *E. coli* strains were investigated using PCR with specific H47 and M primers. PCR products of sequentially related bacteriocins (colicins E2-9, Ia-Ib, U-Y, 5–10) were verified using dideoxy terminator sequencing and amplification primers. Sequence analysis was carried out using Lasergene software (DNASTAR, Inc., Madison, WI, USA).

### Screening for genes encoding virulence factors

All 1181 *E. coli* strains were screened for the presence of genes for 17 different virulence factors (*α*-*hly*, *afaI*, *aer*, *cnf1*, *sfa*, *pap*, pCVD432, *ial*, *lt*, *st*, *bfpA*, *eaeA*, *ipaH*, *iucC*, *fimA*, *stx1*, *stx2* and *ehly*). The primer pair sequences, PCR product lengths and PCR protocols used, were previously described [48-55].

### Statistical analyses

For statistical analysis of the incidence of bacteriocins and virulence factors, standard methods derived from the binomial distribution, including the two-tailed Fisher’s exact test corrected using the Bonferroni correction, were used. *STATISTICA* software, version 8.0 (StatSoft, Tulsa, OK, USA), was used for calculations. Distribution of virulence factors and bacteriocin genes were determined using Correspondence Analysis (CA) and *STATISTICA* version 8.0.

## Availability of supporting data

The data set of 294 colicin gene sequences supporting the results of the article has been deposited in the GenBank/EMBL/DDBJ under accession numbers AB923519 - AB923812.

## Competing interests

The authors declare that they have no competing interests.

## Authors’ contributions

DS designed the study and together with LM wrote the manuscript. LM, BS, JB and LMik performed bacteriocin and virulence testing of *E. coli* strains. LM and SL analyzed the data. MV, AS and VW contributed to isolation and characterization of the bacterial strains and gathered data. All authors read and approved the final manuscript.

## Supplementary Material

Additional file 1: Table S1 Distribution of virulence determinants and bacteriocin genes among 1181 *E. coli* strains isolated from human fecal microflora. Click here for file

Additional file 2: Table S2 DNA Primers used for PCR detection of colicin and microcin encoding genes. Click here for file
