# Supplementary material for: Bacteriocin-encoding genes and ExPEC virulence determinants are associated in human fecal Escherichia coli strains
Source: BMC Microbiol. 2014 Apr 28;14:109. doi: 10.1186/1471-2180-14-109 (PMC4021369; doi:10.1186/1471-2180-14-109)
Supplement: Additional file 1: Table S1 — Distribution of virulence determinants and bacteriocin genes among 1181 E. coli strains isolated from human fecal microflora. [file 1471-2180-14-109-S1.docx]

**Additional file 1: Table S1. Distribution of virulence determinants and bacteriocin genes among 1181 *E. coli* strains isolated from human fecal microflora**

| **Virulence determinant** | | **No. of *E. coli* strains (%)** |  |
| --- | --- | --- | --- |
| aggregative adherence plasmid | pCVD432 | 13 (1.1) |  |
| invasion associated locus | *ial* | 44 (3.7) |  |
| heat-stable enterotoxin | *st* | 8 (0.7) |  |
| heat-labile enterotoxin | *lt* | 7 (0.6) |  |
| intimin | *eaeA* | 26 (2.2) |  |
| bundle-forming fimbriae | *bfpA* | 1 (0.08) |  |
| invasion plasmid H | *ipaH* | 19 (1.6) |  |
| aerobactin synthesis | *aer* | 410 (34.7) |  |
| fimbriae type 1 | *fimA* | 1038 (87.9) |  |
| α-hemolysin | *α-hly* | 91 (7.7) |  |
| afimbrial adhesin I | *afaI* | 78 (6.6) |  |
| aerobactin synthesis | *iucC* | 476 (40.3) |  |
| cytotoxic necrotizing factor | *cnf1* | 44 (3.7) |  |
| S-fimbriae | *sfa* | 233 (19.7) |  |
| P-fimbriae | *pap* | 220 (18.6) |  |
| shiga-toxin 1 | *stx1* | - |  |
| shiga-toxin 2 | *stx2* | - |  |
| enterohemolysin | *ehly* | 9 (0.8) |  |
| **Bacteriocin types** |  | **No. of *E. coli* strains (%)** |  |
| colicin A | | - |  |
| colicin B |  | 44 (3.7) |  |
| colicin D |  | 1 (0.08) |  |
| colicin E1 |  | 128 (10.8) |  |
| colicin E2 |  | 3 (0.3) |  |
| colicin E3 | | 1 (0.08) |  |
| colicin E4 |  | - |  |
| colicin E5 |  | 1 (0.08) |  |
| colicin E6 |  | 3 (0.3) |  |
| colicin E7 |  | 13 (1.1) |  |
| colicin E8 |  | 5 (0.4) |  |
| colicin E9 |  | - |  |
| colicin Ia |  | 201 (17.0) |  |
| colicin Ib |  | 61 (5.2) |  |
| colicin K |  | 25 (2.1) |  |
| colicin L |  | - |  |
| colicin M |  | 101 (8.6) |  |
| colicin N |  | 13 (1.1) |  |
| colicin S4 |  | 11 (0.9) |  |
| colicin U |  | 3 (0.3) |  |
| colicin Y |  | 3 (0.3) |  |
| colicin 5/10 |  | 7 (0.6) |  |
| colicin Js |  | 28 (2.4) |  |
| microcin B17 |  | 75 (6.4) |  |
| microcin C7 |  | 9 (0.8) |  |
| microcin H47 |  | 207 (17.5) |  |
| microcin J25 |  | 2 (0.2) |  |
| microcin L |  | 2 (0.2) |  |
| microcin M |  | 147 (12.4) |  |
| microcin V |  | 188 (15.9) |  |
| **Bacteriocin gene** |  | **No. of *E. coli* strains (%)** |  |
| at least one bacteriocin gene detected |  | 642 (54.4) |  |
| bacteriocin mono-producers* |  | 227 (19.2) |  |
| bacteriocin double-producers** |  | 230 (19.5) |  |
| bacteriocin multi-producers*** |  | 175 (14.8) |  |
| unidentified bacteriocin gene |  | 10 (0.8) |  |
| **Frequent bacteriocin types/combinations** |  | **No. of *E. coli* strains (%)** |  |
| mono-producer - mH47 |  | 58 (4.9) |  |
| mono-producer - Ia |  | 44 (3.7) |  |
| mono-producer - E1 |  | 30 (2.5) |  |
| double-producer - Ia, mV |  | 60 (5.1) |  |
| double-producer - mH47, mM |  | 59 (5.0) |  |

*producers of one bacteriocin type

**producers of two bacteriocin types

***producers of three and more bacteriocin types
