# Supplementary material for: Bacteriocin-encoding genes and ExPEC virulence determinants are associated in human fecal Escherichia coli strains
Source: BMC Microbiol. 2014 Apr 28;14:109. doi: 10.1186/1471-2180-14-109 (PMC4021369; doi:10.1186/1471-2180-14-109)
Supplement: Additional file 2: Table S2 — DNA Primers used for PCR detection of colicin and microcin encoding genes. [file 1471-2180-14-109-S2.docx]

**Additional files 2: Table S2. DNA Primers used for PCR detection of colicin and microcin encoding genes**

| **Colicin and microcin type** | **Primer name** | **5´-sequence-3´** | **Length**  **of PCR product (nt)** | **Reference of primers** |
| --- | --- | --- | --- | --- |
| A | ColA-F | cgtggggaaaagtcatcatc |  |  |
|  | ColA-R | gctttgctctttcctgatgc | 475 | [21] |
| B | colicinB-F | aagaaaatgacgagaagacg |  |  |
|  | colicinB-R | gaaagaccaaaggctataagg | 492 | [26] |
| D | ColD-F | ctggactgctgctggtgata |  |  |
|  | ColD-R | gaaggtgcgcctactactgc | 420 | [21] |
| E1 | colicinE1-F | tgtggcatcgggcgagaata |  |  |
|  | colicinE1-R | ctgcttcctgaaaagcctttt | 649 | [26] |
| E1* | cea2F | ggtggaactggaggtagcaa |  |  |
|  | cea2R | acgtcgttgttgttctgcttcct | 357 | this study |
| E2 | ColE2-F | tgatgctgctgcaaaagag |  |  |
|  | ColE2-R | ttcaaagcgttccctaccac | 409 | [21] |
| E3 | ColE3-F | taagcaggctgcatttgatg |  |  |
|  | ColE3-R | tcggatctggacctttcaac | 413 | [21] |
| E4 | ColE4-F | gaaggctgcatttgatgct |  |  |
|  | ColE4-R | cggatccggacctttaattt | 409 | [21] |
| E5 | ColE3-F | taagcaggctgcatttgatg |  |  |
|  | ColE5-R | ttgaattctcgaatcgtcca | 430 | [21] |
| E6 | ColE6-F | accgaacgtccaggtgtt |  |  |
|  | ColE6-R | tttagcctgtcgctcctgat | 399 | [21] |
| E7 | ColE7-F | gcattctgccatctgaaat |  |  |
|  | ColE7-R | cttctgcccactttctttcg | 431 | [21] |
| E8 | ColE3-F | taagcaggctgcatttgatg |  |  |
|  | ColE8-R | gactgattggcttgtcgtga | 449 | [21] |
| E9 | ColE3-F | taagcaggctgcatttgatg |  |  |
|  | ColE9-R | gacttttctccctccgacct | 418 | [21] |
| Ia | ColIa-F | gcatgcaaatgacgctctta |  |  |
|  | ColIa-R | gaggacgccagttctctgtc | 473 | [21] |
| Ib | ColIb-F | aacgagtgggtcgatgattc |  |  |
|  | ColIb-R | ccttttctgcgctcgtattc | 464 | [21] |
| Js | ColJs-F | tcaaaatgtttgggctcctc |  |  |
|  | ColJs-R | taatctgccctgtcccactg | 254 | [21] |
| K | ColK-F | cagaggtcgctgaacatgaa |  |  |
|  | ColK-R | tccgctaaatcctgagcaat | 469 | [21] |
| L | Col28b(L)-F | tgcatattgaaagcgtcagc |  |  |
|  | Col28b(L)-R | caggttatcccctctcacca | 449 | this study |
| M | ColM-F | gcttaccacttcgcaaaacc |  |  |
|  | ColM-R | gagcgactctccgataatgc | 429 | [21] |
| N | ColN-F | agcttggcgagtatcttgga |  |  |
|  | ColN-R | caacacagccccgaataaac | 401 | [21] |
| S4 | ColS4-F | tatatggcccaactgctggt |  |  |
|  | ColS4-R | cgtaaggacggacacctgtt | 456 | [21] |
| U | ColU-F | tgattgctgcgagaaaaatg |  |  |
|  | ColU-R | tctgacagcctctccctgtt | 485 | [21] |
| Y | ColY-F | gcaggcagaaaagaacaagg |  |  |
|  | ColY-R | cggacgttatttgccttcat | 477 | [21] |
| 5 | Col5-F | cattggcaaaagcgaaatct |  |  |
|  | Col5-R | tgcaactctggaaacaatcg | 443 | [21] |
| 10 | Col10-F | ggttaccggatttcctggat |  |  |
|  | Col10-R | ttctagatgcttggcccact | 448 | [21] |
| B17 | microcin B17-F | tcacgccagtctccattaggtgttggcatt |  |  |
|  | microcin B17-R | ttccgccgctgccaccgtttccaccactac | 135 | [26] |
| C7 | microcin C7-F | cgttcaactgttgcaatgct |  |  |
|  | microcin C7-R | agttgaggggcgtgtaattg | 134 | [21] |
| H47 | microcin H47-F | cactttcatcccttcggattg |  |  |
|  | microcin H47-R | agctgaagtcgctggcgcacctcc | 227 | [26] |
| J25 | microcin J25-F | tcagccatagaaagatataggtgtaccaat |  |  |
|  | microcin J25-R | tgattaagcattttcattttaataaagtgt | 175 | [26] |
| L | microcin  L-F | ggtaaatgatatatgagagaaataacgtta |  |  |
|  | microcin  L-R | tttcgctgagttggaatttcctgctgcatc | 233 | [26] |
| M | M-DS2009-F | cgtttattagcccgggattt |  |  |
|  | M-DS2009-R | gcagacgaagaggcacttg | 166 | this study |
| V | microcin  V-F | cacacacaaaacgggagctgtt |  |  |
|  | microcin  V-R | cttcccgcagcatagttccat | 680 | [26] |

* For colicin E1 detection, an additional primer pair {cea2F (5´-ggtggaactggaggtagcaa-3´) and cea2R (5´-acgtcgttgttgttctgcttcct-3´)} was used to detect sequentially diverse colicin E1 genes.
